# Perception of 3rd year high school students of two schools in Dourados-MS about biotechnology

**DOI:** 10.1186/1753-6561-8-S4-P235

**Published:** 2014-10-01

**Authors:** Carolina Harumi Cavarson, Liliam Silvia Candido, Geovani Fabian Meireles Duarte, Pedro Matheus Rocha, Helder Freitas dos Santos, José Lourenço dos Santos Cunha e Silva, Matheus Fernandes, Gabrielli Cristini Maia, Peceu Magyve Ragagnin de Oliveira

**Affiliations:** 1 Faculty of Biological and Environmental Sciences (FCBA), Federal University of Grande Dourados (UFGD), Dourados, MS, Brazil

## Background

It is known that biotechnology is present in all sectors of contemporary life. Still, it is quite common the divulgation of misconceptions about it, which leads to insecurity and prejudice by the part of society. Thus, to demystify and disseminate biotechnology as science, have been developed by students of Biotechnology from Federal University of Grande Dourados (UFGD), the extension project "Biotechnology for All". Through expository lectures the academics seek to elucidate, clarify doubts and stimulate the interest of students of the 3rd year of high school for this science. So, This study was conducted to evaluate the perception of students of the 3rd year of high school in two schools of Dourados-MS, on topics related to Biotechnology. As a result, it was possible to assess the similarities and differences between the data collected in both institutions.

## Methods

We developed a questionnaire with ten questions addressing general knowledge on topics related to biotechnology, that were applied on students of the 3rd year of high school at a private school and in a public school in Dourados-MS. The evaluation was conducted based on questionnaires completed by 48 students, being 22 private schools (45.8%) and 26 of public (54.2%).

## Results and conclusions

All public school students said they had had contact with biotechnology for a vehicle of communication, and television was identified as the main source of information on the subject for these students. Among students of private education, 20% reported never having had contact with biotechnology by any means of communication. The internet was identified as the main source of information about biotechnology by these students. When asked about the importance of biotechnology to society, 76.5% of respondents said that they considered important but could not explain why, and less than half of the respondents were able to correctly define the meaning of "biotechnology". Respondents were asked to mark among several options the topics that were believed to be related to biotechnology: 86% of public school students and 80% of private school students indicated the correct alternatives. People often mistakenly relate areas such as robotics and computing to biotechnology, probably due to the suffix "technology". At the same time, concepts like transgenics, stem cells and cloning were correctly marked by the most students probably by the large diffusion of these issues on the media. It was found that 75% of students said they knew the meaning of transgenics, although only 39% cited examples such as soybean, oil, and "biscuits". Regarding the acronym GMO (genetically modified organism) 90% of private school students reported not knowing their significance, and 65% of public school students said they knew the meaning of GMOs, but they were not able to cite examples. Most students reported owning a detached position in relation to GMOs and 25% could not answer because they have not enough knowledge about it. In general, respondents showed no basic knowledge about biotechnology, so the project "Biotechnology for All" is an important source of dissemination of this science to society.

